# Vav1 is Essential for HIF-1α Activation via a Lysosomal VEGFR1-Mediated Degradation Mechanism in Endothelial Cells

**DOI:** 10.3390/cancers12061374

**Published:** 2020-05-27

**Authors:** Jaewoo Hong, Yongfen Min, Todd Wuest, P. Charles Lin

**Affiliations:** Center for Cancer Research, National Cancer Institute, National Institutes of Health, Frederick, MD 21704, USA; yongfen.min@nih.gov (Y.M.); todd.wuest1@gmail.com (T.W.)

**Keywords:** Vav1, endothelial cell, HIF-1α, protein degradation, hypoxia, VEGFR

## Abstract

The vascular response to hypoxia and ischemia is essential for maintaining homeostasis during stressful conditions and is particularly critical for vital organs such as the heart. Hypoxia-inducible factor-1 (HIF-1) is a central regulator of the response to hypoxia by activating transcription of numerous target genes, including vascular endothelial growth factor (VEGF). Here we identify the guanine nucleotide exchange factor (GEF) Vav1, a regulator of the small Rho-GTPase and cell signaling in endothelial cells, as a key vascular regulator of hypoxia. We show that Vav1 is present in the vascular endothelium and is essential for HIF-1 activation under hypoxia. So, we hypothesized that Vav1 could be a key regulator of HIF-1 signaling. In our findings, Vav1 regulates HIF-1α stabilization through the p38/Siah2/PHD3 pathway. In normoxia, Vav1 binds to vascular endothelial growth factor receptor 1 (VEGFR1), which directs Vav1 to lysosomes for degradation. In contrast, hypoxia upregulates Vav1 protein levels by inhibiting lysosomal degradation, which is analogous to HIF-1α regulation by hypoxia: both proteins are constitutively produced and degraded in normoxia allowing for a rapid response when stress occurs. Consequently, hypoxia rapidly stabilizes Vav1, which is required for HIF-1α accumulation. This shows that Vav1 is the key mediator controlling the stabilization of HIF1α in hypoxic conditions. With this finding, we report a novel pathway to stabilize HIF-1, which shows a possible reason why clinical trials targeting HIF-1 has not been effective. Targeting Vav1 can be the new approach to overcome hypoxic tumors.

## 1. Introduction

The vascular response to hypoxia is an important mechanism that maintains organ function under stress, which is particularly important for vital organs. The cellular response to hypoxia is regulated by the hypoxia-inducible factor-1 (HIF-1) transcription factor. Hypoxia stabilizes the HIF-1 subunit HIF-1α allowing it to activate transcription and mediate an adaptive response. In endothelial cells, HIF-1 regulates the transcription of vascular endothelial growth factor (VEGF), and autocrine VEGF signaling is essential for endothelial cell survival and vascular homeostasis [1,2]. VEGF stimulates cellular responses by binding to the cell surface receptors, VEGFR1 and VEGFR2, on the vascular endothelium. VEGFR2 appears to mediate almost all of the known cellular responses to VEGF [3], whereas VEGFR1 is considered to be inhibitory by acting as a decoy receptor, competing with VEGFR2 for binding to VEGF [3]. 

Angiogenesis, the formation of blood vessels, is an adaptive response of tissues to hypoxia. The transcription factor hypoxia-inducible factor-1 (HIF-1) is considered a central regulator of hypoxia. HIF-1α is stabilized under hypoxia, and thereby mediates adaptive responses to hypoxia by activating transcription of numerous angiogenic genes, such as VEGF, and inducing angiogenesis. Likewise, loss of HIF-1α in endothelial cells disrupts a hypoxia-driven VEGF autocrine loop necessary for tumorigenesis [2]. Endothelial expression of HIF-1 regulates endogenous VEGF expression and autocrine VEGF signaling is essential for endothelial cell survival [1]. As a result, HIF-1 induces angiogenesis and enhances endothelial survival, in so doing restoring tissue oxygen homeostasis under ischemic conditions.

VEGF is an important signaling protein involved in various activities in vascular biology [3]. It stimulates cellular responses by binding to cell surface receptors, VEGFR1 and VEGFR2, on vascular endothelium. VEGFR2 appears to mediate almost all of the known cellular responses to VEGF [3]. The function of VEGFR1 is less well defined, although it is thought to modulate VEGFR2 signaling. The deletion of VEGFR1 in mice results in embryonic lethality due to overgrowth of endothelial cells, leading to disorganization and dysfunction of the vasculature [3]. VEGF-A, the major factor for angiogenesis, binds to two receptor tyrosine kinases, VEGFR-1 and VEGFR-2, and regulates endothelial cell proliferation, migration, vascular permeability, secretion and other endothelial functions. VEGFR-2 exhibits a strong RTK activity towards pro-angiogenic signals, whereas the soluble VEGFR-1 functions as an endogenous VEGF inhibitor [4,5]. However, not much is known about the regulatory mechanisms controlling the differential expression of VEGFR1. Perhaps a net balance in the production of VEGFR1 isoforms determines vessel growth and regression. These findings suggest the possibility that the primary function of VEGFR1 is as a negative regulator of vascular development. The other possible mechanism may be that VEGFR1 acts as a decoy receptor, competing with VEGFR2 for binding to VEGF. 

The levels of proteins within cells are determined not only by synthesis, but also by degradation. Many rapidly degraded proteins function as regulatory molecules, such as transcription factors. The rapid turnover of these proteins is necessary to allow their levels to change quickly in response to external stimuli. In eukaryotic cells, two major pathways—the ubiquitin-proteasome pathway and lysosomal proteolysis—mediate protein degradation [6]. The first pathway of selective protein degradation in eukaryotic cells uses proteasome that targets cytosolic and nuclear proteins for rapid proteolysis [7]. The other pathway of protein degradation in eukaryotic cells involves the uptake of proteins by lysosomes. Lysosomes are membrane-enclosed organelles that contain digestive enzymes, including several proteases [8]. They have several roles in cell metabolism, including the digestion of extracellular proteins taken up by endocytosis as well as the gradual turnover of cytoplasmic organelles and cytosolic proteins [6,9].

Vav1 (also known as Vav) is a guanine nucleotide exchange factor (GEF) that activates small Rho GTPase. Rho GTPase is well known for its functions in the regulation of cytoskeleton arrangement, cell motility and cell-cell adhesion. The Vav family has three members in vertebrates with Vav1 mostly restricted to hematopoietic cells, being found from the pluripotent stem cells to most mature stages of the lymphoid and myeloid-erythroid lineages [10,11]. Vav1 was initially identified as an oncogene capable of transforming NIH3T3 cells, and subsequently, it was found to be an important signal transducer with a pivotal role in hematopoietic cell activation, cell growth and differentiation [10,11,12]. Mice without Vav1 are viable, fertile and grossly normal except with partial blockade in lymphocyte development [13,14,15]. Since hematopoietic cells and endothelial cells share a common progenitor, it is no surprise that Vav1 is also detected in vascular endothelium using Vav1 promoter-driven Cre reporter mice [16]. However, its function in the vascular system is completely unknown.

In this study, we hypothesized that Vav1 protein is upregulated in hypoxia, which may contribute to the stress mechanism in hypoxia by HIF-1 regulation. We found the mechanism of how Vav1 is controlled in hypoxia, which may contribute to the understanding of tumor progressions in hypoxia, such as aggressive hypoxic tumors.

## 2. Results

### 2.1. Vav1 Is Present in the Endothelium and Is Controlled by Lysosomal Protein Degradation

**Figure 1:**
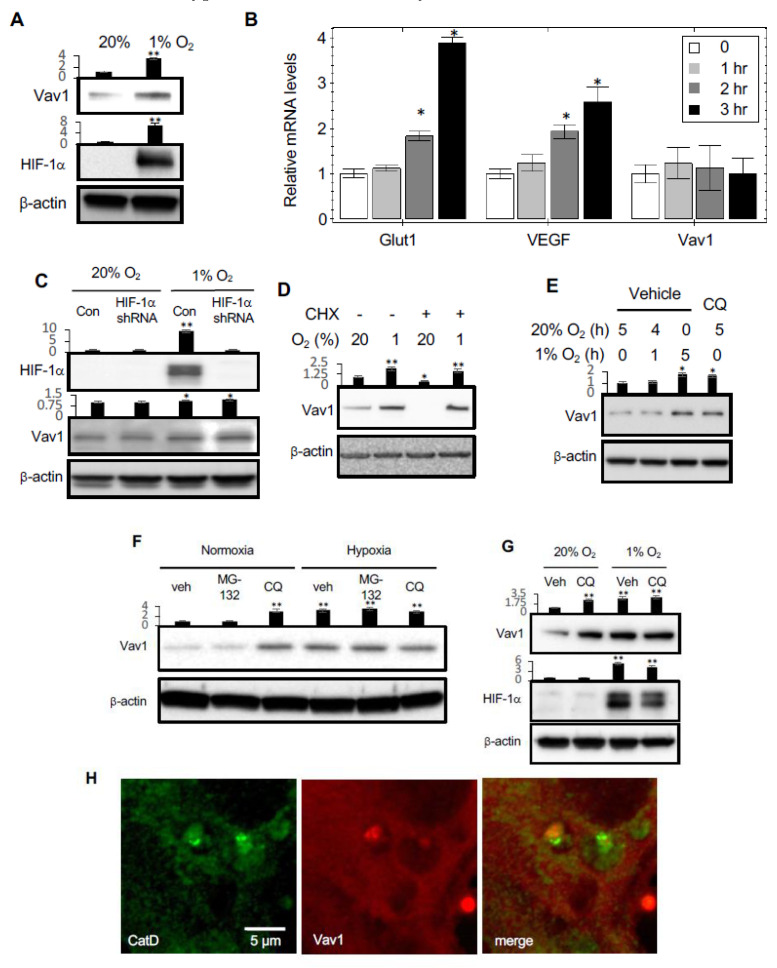
Hypoxia upregulates Vav1 through inhibition of lysosome-mediated protein degradation. (**A**) Human umbilical vein endothelial cells (HUVEC)s were cultured either in 20% or 1% O_2_ for 5 h, followed by Western blot analysis for the indicated proteins. (**B**) qPCR analysis of Glut1, VEGF and Vav1 in HUVECs cultured in 1% O_2_ for 0, 1, 2, or 3 h. * *p* < 0.05 compared to corresponding time 0 in each group (mean ± SD). (**C**) The levels of Vav1 and HIF-1α were measured by Western blot of HUVECs transduced with HIF-1α or scramble shRNA expressing Lentivirus for 24 h. (**D**) HUVECs were incubated in normoxic and hypoxic environments for 5 h in the presence or absence of 10 μM cycloheximide. Protein levels were measured by Western blot from the total lysate. (**E**) HUVECs were incubated in 20% O_2_ for 5 h, incubated in 20% O_2_ for an hour and then moved to 1% O_2_ for 4 h, incubated in 1% O_2_ for 5 h. The level of Vav1 in the lysate was compared to HUVECs incubated in 20% O_2_ in the presence of chloroquine (CQ) at 50 μM for 5 h in order to identify whether Vav1 is affected by lysosomal inhibition. (**F**) HUVEC were incubated in normoxic or hypoxic conditions for 4 h in the presence of either vehicle control, 5 μM of MG-132, or 50 μM of chloroquine (CQ). The total lysate was subjected to Western blotting to measure Vav1 protein levels. (**G**) Western blot analysis of Vav1 levels in HUVECs cultured in either 20% or 1% O_2_ in the absence or presence of Bafilomycin A (Baf A) at 100 nM for 5 h. (**H**) Immunofluorescent staining for Vav1 (red) and Cathepsin D (green) in HUVECs were imaged by an LSM780 confocal microscope. Each experiment was repeated at least ten times and representative images are shown. Mean ± SD, * *p* < 0.05, ** *p* < 0.01. The whole western blot images please find in Figure S1.

Although Vav1 is largely considered to be a hematopoietic-specific protein [10,11,12], blood cells and endothelial cells are derived from a common progenitor, and a genetic tracing study identified Vav1 in the endothelium [16]. We found that incubation of human umbilical vein endothelial cells (HUVEC) in hypoxic conditions for 5 h caused an increase in Vav1 protein levels (Figure 1A). To determine whether this increase occurs at the protein or mRNA level, the mRNA levels of Vav1 were also measured under hypoxic conditions for 1, 2 or 3 h. Unlike the hypoxia-response element (HRE) genes regulated by the transcription factor, HIF1α, such as GLUT-1 and VEGF, there was no increase in Vav1 mRNA levels under hypoxic conditions (Figure 1B). To determine whether Vav1 is affected by HIF1α, we knocked down HIF1α using shRNAs in HUVEC. Under hypoxic conditions, there was no effect of HIF1α knockdown on Vav1 levels compared to control cells (Figure 1C). Thus, the increase in Vav1 under hypoxia is not mediated by HIF1α.

To investigate the mechanism of regulation of Vav1 levels in hypoxia, HUVECs were cultured in the presence of cycloheximide to suppress nascent protein synthesis from mRNA, followed by sequential incubation of the cells in normoxia and hypoxia for 5 h, to analyze the specific effect of protein degradation on Vav1 levels. In normoxia, the addition of cycloheximide led to a significant reduction of Vav1 protein levels compared to vehicle-treated cells. This indicates that under normal conditions, Vav1 protein is being continuously synthesized and degraded. In contrast, in hypoxia, the presence of cycloheximide resulted in the increase of Vav1 protein levels both with or without the treatment of cycloheximide (Figure 1D). These findings imply that under hypoxic conditions, Vav1 protein levels are increased by the inhibition of its degradation. 

To determine whether Vav1 degradation is mediated through the proteasomal or lysosomal pathway, we cultured HUVECs in normoxia in the presence of lactacystin to inhibit the proteasomal pathway, or in the presence of chloroquine, a lysosomal inhibitor. The addition of chloroquine resulted in a significant increase in Vav1, to levels close to those observed when cells were cultured in hypoxia for several hours (Figure 1E). In contrast, treatment with the proteasomal inhibitor, MG-132, did not significantly affect Vav1 levels (Figure 1F). 

To corroborate the role of lysosomes in Vav1 degradation, we measured the levels of Vav1 and HIF1α in the presence or absence of chloroquine also in hypoxia. We confirmed that blocking lysosomal activation led to increased Vav1 in normoxia, while HIF1α was not affected. Chloroquine had no additional effect on the upregulation of Vav1 in hypoxia (Figure 1G), suggesting that the lysosomal degradation of Vav1 is already inhibited. We examined if Vav1 was present in lysosomes by performing immunofluorescent staining with antibodies against Vav1 and cathepsin D (CatD), a lysosomal marker, in HUVECs. Vav1 protein co-localized in lysosomes with CatD (Figure 1H). Collectively, these results suggest that lysosomes mediate Vav1 degradation in normoxia, and hypoxia blocks this protein degradation, leading to Vav1 accumulation.

### 2.2. VEGFR1-Bound Vav1 is Carried to Lysosomes

**Figure 2:**
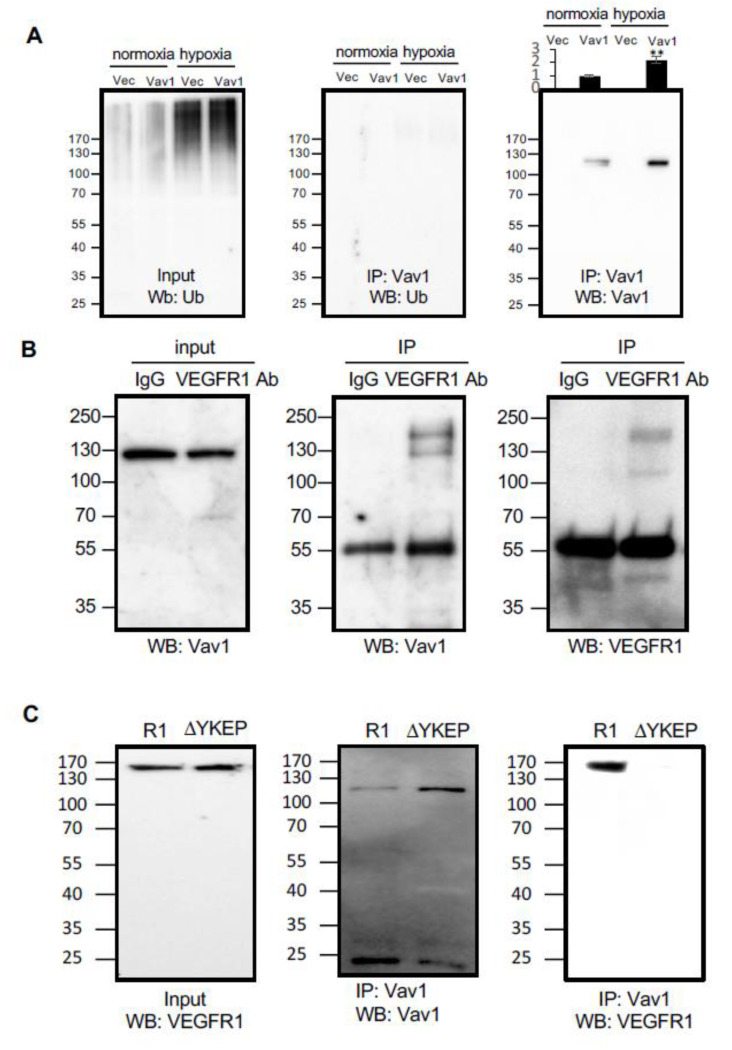
Vav1 binds to vascular endothelial growth factor receptor 1 (VEGFR1). (**A**) Either Flag-tagged Vav1 or empty vector control were transduced into HUVEC cells and incubated under normoxic or hypoxic conditions. Cell lysates were subjected to Vav1 immunoprecipitation and subsequent Western blot analysis for ubiquitin (middle panel) or Vav1 as a control (right panel). Ubiquitin levels were also detected in both control and Vav1 cells exposed to hypoxia from the input of the immunoprecipitation (left panel). (**B**) VEGFR1 and Vav1 were immunoprecipitated by anti-VEGFR1 antibody or control IgG from the cell lysate of HUVEC cells. Vav1 was probed from the input lysate (left) and pulldown product (center). VEGFR1 was detected from the pulldown product (right). (**C**) Overexpressed wild type (R1) or YKEP motif deleted (ΔYKEP) VEGFR1 were co-IPed with Vav1 antibody. The input was probed with VEGFR1 antibody (left). The pulldown product was probed against Vav1 (center) or VEGFR1 antibody (right). Mean ± SD, * *p* < 0.05, ** *p* < 0.01

**Figure 3:**
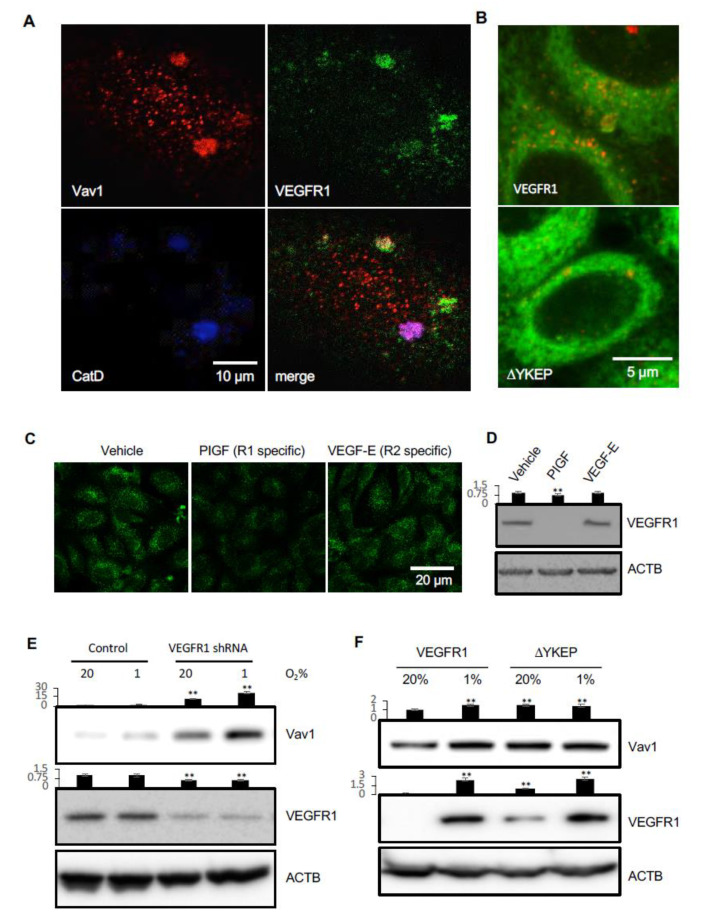
VEGFR1 carries Vav1 to lysosomes for degradation. (**A**) HUVECs were immunostained for Cathepsin D, Vav1 and VEGFR1, and analyzed by confocal microscopy. (**B**) HeLa cells were co-transfected with expression vectors for Vav1 and wild type VEGFR1 or the Vav1 binding domain deleted construct, ΔYKEP. Cells were stained with Lamp2 (red) and Vav1 (green). (**C**) HUVECs were stimulated with either PlGF or VEGF-E at 50 ng/mL for 5 h, followed by staining with antibodies for Vav1 (green) and imaged under confocal microscopy. (**D**) HUVECs were stimulated with either PlGF or VEGF-E at 50 ng/mL for 5 h, followed by Western blot to detect VEGFR1 levels. (**E**) HUVECs were infected with lentiviral vectors carrying scrambled or VEGFR1 shRNAs and cultured in either 20% O_2_ or 1% O_2_ incubators. Cell lysates were analyzed by Western blot for Vav1 and VEGFR1. (**F**) HUVECs were transfected with expression vectors for Vav1 with either VEGFR1 or ΔYKEP VEGFR1. The levels of Vav1 and VEGFR1 were assessed by Western blot. Mean ± SD, ** *p* < 0.01. The whole western blot images please find in Figure S2.

Protein ubiquitination is a sorting signal that targets proteins to multivesicular bodies (MVBs) for degradation. To investigate the mechanism by which Vav1 localizes to lysosomes, we first examined whether Vav1 is ubiquitinated in HUVECs. Hypoxia increased total protein ubiquitination in cells transfected with either the vector control or a Vav1 overexpressing construct (Figure 2A). However, there was no detectable ubiquitination of Vav1 in either normoxic or hypoxic groups (Figure 2A). This led us to speculate that there may be a carrier that transports Vav1 to lysosomes for degradation. Since receptor tyrosine kinases (RTKs) have been shown to undergo lysosomal-mediated degradation after activation [17], we first focused on the possibility of RTKs carrying Vav1 to lysosomes. Among RTKs, VEGFRs are abundant in endothelial cells [17]. The SH2 domain of Vav1 is known to recognize the four-amino acid-binding motif: Y-X-E-P [18]. Sequence analysis revealed the presence of this motif in VEGFR1 (aa990–993), but not in other VEGFRs. Next, we investigated if VEGFR1 transports Vav1 to lysosomes for degradation. To test this, we co-transfected HUVECs with expression vectors for Vav1 and VEGFR1, followed by immunofluorescent staining for these two proteins and for Cathepsin D, a lysosomal marker. The results show the co-localization of Vav1 and VEGFR1 in lysosomes (Figure 3A). We then tested whether binding with VEGFR1 is required for Vav1 translocation to lysosomes. HUVECs were co-transfected with expression vectors for Vav1 along with WT VEGFR1 or the YKEP deletion mutant, followed by staining for Vav1, VEGFR1 and Cathepsin D. As predicted, disruption of VEGFR1 binding reduced Vav1 localization in lysosomes (Figure 3B). The data suggest that VEGFR1 binds to Vav1 and transports it to lysosomes.

The above findings led us to examine if ligand-binding to endogenous VEGFR1 induces Vav1 degradation. We stimulated HUVECs with PlGF, a VEGFR1-specific ligand, or VEGF-E, a VEGFR2-specific ligand, for 30 min. Activation of VEGFR1 induced Vav1 degradation, while activation of VEGFR2, which we predicted would not bind to Vav1, did not change the levels of Vav1 compared to controls (Figure 3C,D). Moreover, knockdown of VEGFR1 using shRNA in HUVECs increased the levels of Vav1 in both normoxia and hypoxia, consistent with it inducing Vav1 degradation (Figure 3E). This is in line with our recent finding that ectopic expression of the VEGFR1 YKEP deletion mutant also led to an increase of Vav1 compared to VEGFR1 transfected cells in normoxia (Figure 3F) [19]. Hypoxia increased Vav1 levels in VEGFR1 transfected cells, but the levels of Vav1 in YKEP transfected cells remained the same in normoxia and hypoxia (Figure 3F). Collectively, these data suggest that VEGFR1 carries Vav1 to lysosomes, and activation of the receptor induces Vav1 degradation. 

### 2.3. Vav1 is Essential for HIF-1α Stabilization via Regulation of p38 Activation in Response to Hypoxia

**Figure 4:**
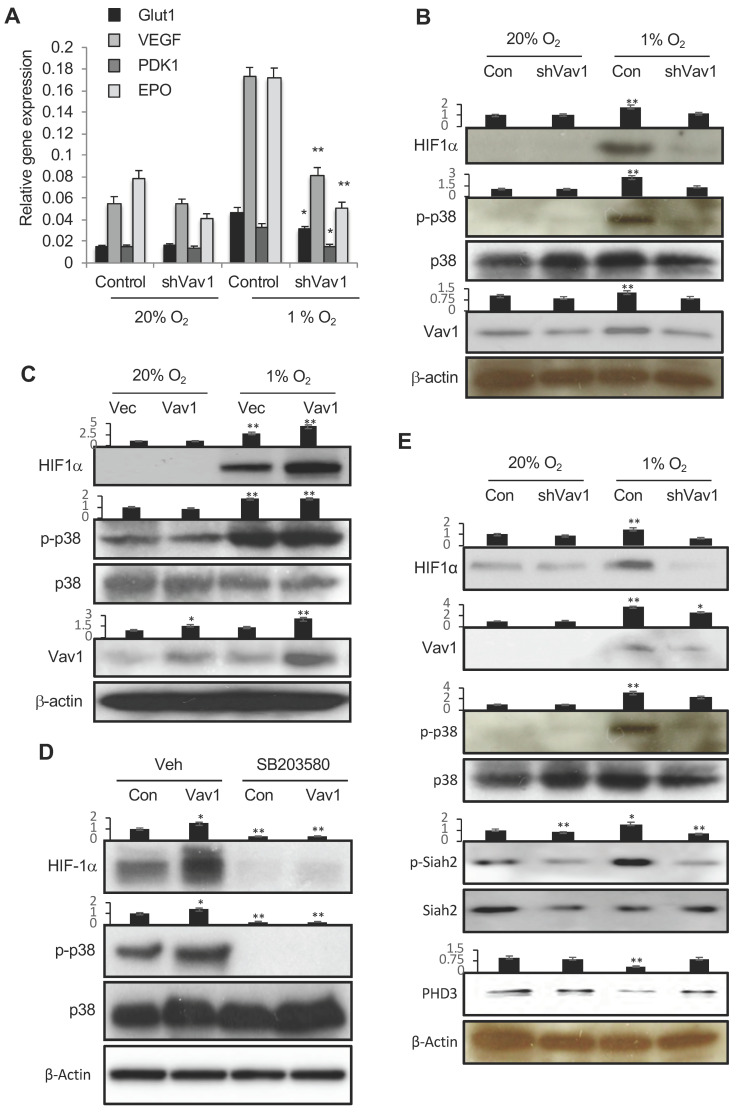
Vav1 regulates HIF1α via p38 MAPK. (**A**) HUVECs were transfected with a control vector or shVav1 vector for 24 h, followed by incubation either in normoxia or hypoxia for another 24 h. HIF-1 target gene expression was analyzed by qRT-PCR. * *p* < 0.05 and ** *p* < 0.01 compared to corresponding control transfected cells in hypoxia (mean ± SD). Each experiment was performed in triplicate and repeated three times. (**B**) Control vector- or shVav1 vector-transfected HUVECs were incubated under normoxia or hypoxia conditions for 24 h. The levels of phosphor-p38, HIF-1α, p38 and Vav1 were analyzed by Western blot. (**C**) Identical procedures and measurements as in (**B**) except the cells were transfected with a control vector or Vav1 expression vector. (**D**) Control or Vav1 expression vector-transfected HUVECs were incubated in the absence or presence of SB203580 at 10 μM in hypoxia for 24 h. The levels of HIF-1α, p38 and phospho-p38 were analyzed by Western blot. (**E**) Control vector- or shVav1 vector-transfected HUVECs were incubated under normoxic or hypoxic conditions for 24 h. The levels of phospho HIF-1α, Vav1, P-p38, p38, P-siah2, Siah2 and PHD3 were analyzed by Western blot. Mean ± SD, * *p* < 0.05, ** *p* < 0.01. The whole western blot images please find in Figure S3.

HIF-1 is a central regulator of the response to hypoxic conditions. Hypoxia stabilizes HIF-1α and activates transcription of numerous target genes. To explore the link between Vav1 and HIF-1, we knocked down Vav1 in cultured HUVECs, followed by incubation of the cells in normoxic or hypoxic conditions. The expression of HIF-1 target genes, including *VEGF, EPO, Glut1* and *PDK1*, were analyzed by qPCR. The knockdown of Vav1 in human endothelial cells significantly impaired the expression of HIF-1 target genes under hypoxic conditions (Figure 4A). These data reveal an essential role for Vav1 in hypoxia-induced HIF-1α stabilization in endothelial cells.

Hypoxia activates the MAPK p38 [20], and p38 stabilizes HIF-1α [21]. Therefore, to dissect the signaling mechanism by which Vav1 regulates HIF-1^α^ accumulation, we first investigated the role of Vav1 in p38 activation. Consistent with reported data, hypoxia induced p38 phosphorylation and accumulation of HIF-1α protein in HUVECs. However, these responses were largely blocked after the knockdown of Vav1 (Figure 4B). Conversely, overexpression of Vav1 further increased hypoxia-induced p38 phosphorylation as well as HIF-1α protein accumulation during hypoxia (Figure 4C). Thus, Vav1 is required for both p38 phosphorylation and HIF-1α accumulation in hypoxia. To determine the role of p38 in Vav1-mediated HIF-α accumulation, we ectopically expressed Vav1 in HUVECs and incubated the cells in hypoxia in the presence or absence of a p38-specific inhibitor, SB203580. The blocking p38 activation blunted HIF-1α accumulation under both control and Vav-1 overexpression conditions (Figure 4D). Together, these data imply that p38 activation acts downstream of Vav1 for HIF-1α accumulation upon hypoxic stimulation. The ubiquitin ligase Siah2 regulates the stability of prolyl hydroxylase-3 (PHD3) that targets HIF-1α for degradation [22]. Vav1 knockdown in hypoxia also abrogated the phosphorylation of Siah2, which led to increased levels of PHD3 that likely contributes to HIF1α degradation (Figure 4E). These data support Vav1 acting upstream of p38 and being essential for hypoxia-mediated activation of p38. Without Vav1, hypoxia is unable to activate p38, preventing the subsequent activation of Siah2 and PHD3 degradation, necessary for HIF-1α accumulation.

### 2.4. Hypoxia Inhibits the Degradation of VEGFR1 and Vav1

**Figure 5:**
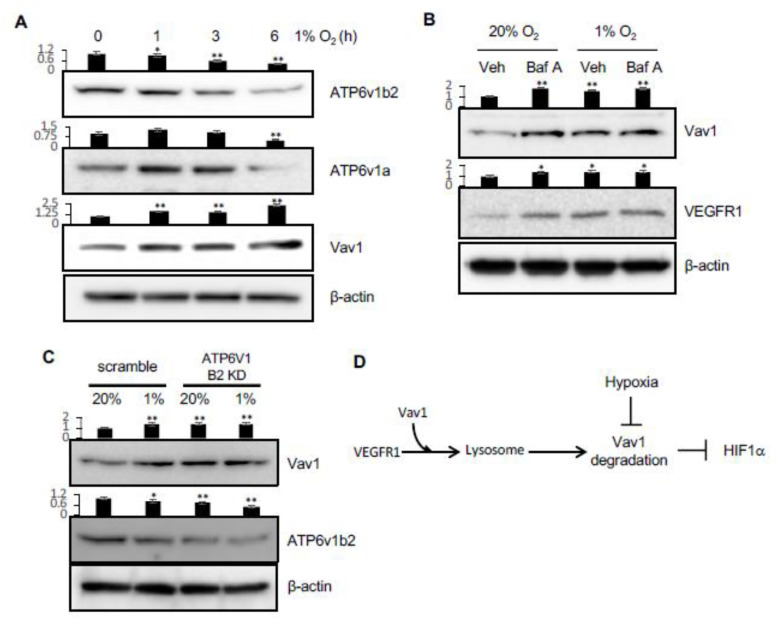
Hypoxia regulates lysosomal activity via v-ATPase and Vav1 levels. (**A**) HUVEC cells were incubated in hypoxia for 0, 1, 3 or 6 hours. Vav1, ATP6v1a, and ATP6v1b2 were measured in the total lysates. (**B**) HUVEC were incubated in normoxia or hypoxia for 5 h in the presence or absence of 100 nM of Bafilomycin A (Baf A). Vav1 and VEGFR1 were measured by Western blot of the total lysates. (**C**) HUVEC were transduced with ATP6v1b2 shRNA-expressing lentivirus or scrambled shRNA virus for 72 h, and then incubated in normoxia or hypoxia for 5 h. Vav1 and ATP6v1b2 levels were measured by Western blot of the total lysate. (**D**) VEGFR1 carries Vav1 to the lysosome in the cell. Vav1 is degraded in the lysosome, which downregulates HIF1α. Hypoxia blocks the lysosomal degradation. Mean ± SD, * *p* < 0.05, ** *p* < 0.01. The whole western blot images please find in Figure S4.

In our recent study, we showed that lysosomal activity is downregulated in hypoxic conditions, which leads to the upregulation of receptor tyrosine kinase molecules [19]. This inhibition of lysosomal activity is caused by a decrease in the levels of v-ATPase components, which inactivates lysosomes (Figure 5A) [19]. Since VEGFR1 is a receptor tyrosine kinase, we tested the levels of Vav1 and VEGFR1 after the inhibition of v-ATPase in lysosomes by Bafilomycin A in both normoxia and hypoxia. As predicted, VEGFR1 was upregulated upon Bafilomycin A treatment in normoxic conditions. However, under hypoxic conditions, when lysosomal activity is decreased, Bafilomycin A was unable to further upregulate VEGFR1 levels. This pattern was also seen for Vav1, which further supports a role for the lysosome, and potentially also VEGFR1, in regulating Vav1 levels (Figure 5B). To test this more directly, we knocked down one of the components of the v-ATPase. This led to an increase in Vav1 levels in normoxia, with no further increase in hypoxia (Figure 5C). These data imply hypoxia inhibits the degradation of VEGFR1 and Vav1 by inactivating the lysosomes, as we recently reported [19].

## 3. Materials and Methods

### 3.1. Experimental Designs

In order to determine which level of Vav1 is controlled, we measured the protein and mRNA level; the protein level was measured when the nascent protein synthesis was blocked by cycloheximide. The pathway of Vav1 proteolysis was determined by chloroquine for lysosomal inhibition and MG-132 for proteasomal inhibition. The location of the Vav1 molecule was determined by confocal microscopy. Following the analysis of the protein sequence of Vav1, the interaction of Vav1 and VEGFR1 was determined by immunoprecipitation as well as the mutant study of the candidate interaction site from VEGFR1. Since we hypothesized that VEGFR1 is the carrier of Vav1 for the lysosomal degradation pathway, we triggered VEGFR1 using PlGF, which led to the degradation of VEGFR1 and Vav1. Since we observed that Vav1 controls HIF1α, we studied the mechanism. Since p38 MAPK is affected by hypoxia and HIF1α, we measured the p32/Siah2/PHD3 mechanism after controlling Vav1 genetically. Along with our previous finding, that lysosomal activity is controlled by hypoxia through the mTORc1/TFEB/V-ATPase pathway [19], we validated that Vav1 degradation in hypoxia is affected by this pathway. In order to prove this, we measured the level of V-ATPase component molecules in HUVECs incubated in hypoxia.

### 3.2. Cell Culture and Reagents

HUVECs (Lonza, Walkersville, MD, USA) were cultured with EGM-2 medium (Lonza) and maintained at 37 °C with 5% CO_2_. Hypoxia was performed by incubating cells in an incubator with 1% O_2_ (Thermo, Middletown, VA, USA). Human PlGF and VEGF-E were purchased from ProSpec (East Brunswick, NJ, USA). Cycloheximide and chloroquine were purchased from Tocris (Bristol, UK), bafilomycin-A (BafA) was from Sigma (St. Louis, MO, USA). HeLa cells were obtained from ATCC (Manassas, VA, USA) and cultured in 10% FBS (ThermoFisher Scientific, Waltham, MA, USA) containing DMEM (ThermoFisher Scientific).

### 3.3. Transfection and Lentiviral Transduction

Lentiviral control, Vav1 shRNA, VEGFR1 shRNA, and ATP6v1b2 constructs were obtained from Sigma (St. Louis, MO, USA). Constructs were prepared as lentivirus to transduce knockdown or overexpression. Briefly, lentiviral vectors were co-transfected with VSV.G and envelope vectors into 60% confluent 293T cells in 10-cm culture plates using Fugene HD (Promega, Madison, WI, USA). After three days of incubation, the culture supernatant was collected and concentrated to 500 μL using Lenti-X concentrator (Takara, Kusatsu, Japan). 8 μg/mL of polybrene and 10 μL of the concentrated virus was added to HUVEC and then incubated for 48 h or more for transduction.

### 3.4. Western Blot and Immunoprecipitation

For hypoxic treatment, HUVECs were incubated under either 20% O_2_ or 1% O_2_ for 24 h. The levels of HIF-1α, Siah2, pSiah2, PHD3, p38 and phospho-p38 were analyzed by Western blot using specific antibodies (Cell Signaling, Danvers, MA, USA). SB 203580 (Cell Signaling) at 10 μM was used to inhibit p38 phosphorylation. 

For immunoprecipitation, HeLa cells were lysed with lysis buffer (Cell Signaling) and immunoprecipitated with antibodies against Flag (Sigma, St. Louis, MO, USA) or VEGFR1 (Genetex, Irvine, CA, USA) overnight, followed by protein A/G magnetic beads (Pierce, Waltham, MA, USA). The membranes were probed with antibodies against Vav1 (EMD Millipore, Billerica, MA, USA), Flag, HIF-1α (BD Biosciences, San Jose, CA, USA), VEGFR1 (Abcam, Cambridge, UK) and Ubiquitin (Cell Signaling). Western blot images were quantified by using densitometry [23]. 

### 3.5. Immunofluorescent Staining

Cells were stained with 4% paraformaldehyde for 30 min. Cells were probed with 1:200 diluted primary antibodies and 1:500 diluted fluorescent secondary antibodies. Vav1 antibody was obtained from Genetex (Irvine, CA, USA), VEGFR1 and Cathepsin D antibodies were obtained from ThermoFisher (Waltham, MA, USA), Lamp2 antibody was purchased from DHSB (Iowa City, IA, USA). At least ten images were analyzed from each group with a LSM780 confocal microscope (Zeiss, Oberkochen, Germany) and the representative images were selected.

### 3.6. Real-Time RT-PCR Analysis

Real-time RT-PCR was performed using total RNA isolated on RNeasy Quick spin columns (QIAGEN, CA). One μg of total RNA was used to perform a reverse transcriptase-polymerase chain reaction (RT-PCR) using iScript supermix (Biorad, Hercules, CA, USA). The sequence of PCR primers used are: Vav1, 5′-CAACCTGCGTGAGGTCAAC-3′ and 5′-ACCTTGCCAAAATCCTGCACA-3′; VEGF, 5′-TGTACCTCCACCATGCCAAGT-3′ and, 5′-CGCTGGTAGACGTCCATGAA-3′; PDK1, 5′-ACCAGGACAGCCAATACAAG-3′, and 5′-CCTCGGTCACTCATCTTCAC-3′; Glut1, 5′-ACGCTCTGATCCCTCTCAGT-3′ and 5′-GCAGTACACACCGATGATGAAG-3′; EPO, 5′-ACCAACATTGCTTGTGCCAC-3′ and 5′-TCTGAATGCTTCCTGCTCTGG-3′. Values are expressed as fold increases relative to the reference sample (untreated control) and analyzed with CFX manager (Biorad). All primers were purchased from Sigma. 

### 3.7. Statistical Analysis

All statistical analyses were carried out using Prism 6 (La Jolla, CA, USA). Quantitative variables were analyzed by *t*-test, one-way ANOVA test. All statistical analysis was two-sided, and *p* < 0.05 was considered statistically significant.

## 4. Conclusions

The vascular response to hypoxia is a powerful mechanism to maintain organ function by reducing the negative effects of oxygen deprivation. Thus, the identification of molecular mediators that regulate vascular homeostasis is of great importance. This study identifies Vav1 as a key regulator of the vascular response to hypoxia. We find that Vav1 is continually produced and degraded in normoxic conditions by interaction with VEGFR1 and trafficking to the lysosomes. Hypoxia somehow blocks this degradation mechanism, leading to Vav1 accumulation, which subsequently leads to HIF-1α accumulation. 

Vav1 is largely considered to be a hematopoietic-specific protein [10,11,12]. The Vav1 promoter-driven Cre mice are commonly used for specific gene deletion in hematopoietic cells. However, the current study confirms expression of Vav1 in endothelial cells, which is in agreement with a genetic tracing study indicating Vav1 in endothelium [16], as well as the notion that endothelial cells and blood cells are derived from a common progenitor, and they often share common mediators and pathways. This finding raises a concern regarding specificity when using Vav1-Cre mice for gene deletion, specifically in hematopoietic cells.

Endothelial-specific deletion of HIF-1 disrupts a hypoxia-driven VEGF autocrine loop [2]. The endogenous production of VEGF in endothelial cells and cell-autonomous activity is crucial for vascular homeostasis. In the absence of autocrine VEGF signaling, endothelial cells undergo apoptosis [1]. This phenotype is manifested without detectable changes in the total levels of VEGF and cannot be rescued by exogenous VEGF [1]. Our data demonstrate that Vav1 is essential for HIF-1α accumulation in hypoxia in endothelial cells. Thus, in the absence of Vav1, the endothelium is unable to achieve HIF-1 activation and induction of VEGF, likely leading to a significant increase in endothelial apoptosis under stress. 

Hypoxia activates p38 MAPK [20], and p38 stabilizes HIF-1α [21]. In T cells, Vav1 acts as a point of integration of signal transduction for receptor-mediated p38 activation [24]. Consistent with these findings, we show that hypoxia induces p38 phosphorylation and HIF-1α accumulation in endothelial cells, which is dependent on Vav1. Without Vav1, hypoxia fails to activate p38, thereby interrupting the pathway of HIF-1α accumulation. p38 phosphorylates Siah2, which increases Siah2-mediated degradation of PHD3, thus preventing HIF-1α degradation [25]. Our data suggest that Vav1 is upstream of p38 and is essential for hypoxia-mediated activation of p38. Without Vav1, hypoxia is unable to activate p38, preventing the subsequent activation of Siah2 and PHD3 degradation, necessary for HIF-1α accumulation. 

Vav1 is continually produced in endothelial cells and has a high turnover rate due to lysosomal-mediated degradation. These findings reveal that the regulation of Vav1 is analogous to HIF-1α regulation. Both proteins are constitutively produced, and both are continually degraded via lysosomal (Vav1) and proteasomal (HIF-1α) pathways under normoxia. Hypoxia stabilizes Vav1, and Vav1 is essential for HIF-1α accumulation. Together, these two proteins are key mediators of the vascular response to hypoxia.

It has been reported that Vav1 is targeted to lysosomes by interaction with the cytoplasmic chaperone Hsc70 for degradation in pancreatic tumor cells [26]. In this study, we found that Vav1 is transported to lysosomes by another carrier protein, VEGFR1, in endothelial cells. VEGFR1 is a receptor tyrosine kinase molecule that is known to be degraded by lysosomal proteolysis [27]. Vav1 binds at the binding motif of Y-K-E-P to VEGFR1, and knockdown of VEGFR1 inhibits Vav1 degradation, and conversely activation of VEGFR1 increases Vav1 degradation. VEGFR1 is known as an inhibitory receptor for VEGF, which promotes angiogenesis. Thus, our findings suggest a potential new mechanism by which VEGFR1 inhibits VEGF-mediated angiogenesis: activation of this receptor induces Vav1 degradation, a GEF protein for small RhoGTPase, and thus plays a negative role in cell motility and angiogenesis.

Hypoxia is a condition in which the body or a region of the body is deprived of adequate oxygen supply at the tissue levels [28]. It is a common stress associated with various pathological disorders such as cancer and affects many aspects of cellular and molecular activities, as well as therapeutic responses. Hypoxic tumor cells have invasive and migratory behavior [28]. Furthermore, hypoxic tumor cells are less responsive to chemotherapy and not easy to treat in clinical data. Our recent finding proved that hypoxia downregulates lysosomal activity [19]. Furthermore, through this study, we report why HIF-1 control is not simply controlled by hypoxia by itself. These findings may provide a molecular explanation for the poor therapeutic targeting of HIF in several clinical trials, and for the observation that hypoxic tumors are often aggressive and resistant to therapy. Hopefully, controlling HIF-1 through Vav1 and lysosomal activity may suggest a new therapeutic approach to hypoxic tumors.

In summary, this study reports a protective role of Vav1 in vascular biology. Vav1 controls HIF-1α stabilization through the p38/Siah2/PHD3 pathway. In normoxic condition, Vav1 binds to VEGFR1, which carries Vav1 and donates to lysosomes for proteolysis. In contrast, hypoxia upregulates Vav1 protein by inhibiting lysosomes, which is analogous to HIF-1α regulation by hypoxia: both proteins are constitutively produced and degraded in normoxia, allowing homeostasis. Consequently, hypoxia rapidly stabilizes Vav1, which is required for HIF-1α accumulation. Our study shows that Vav1 is the key mediator controlling the stabilization of HIF1α in hypoxia. With this finding, we report a novel pathway to stabilize HIF-1, which explains why clinical trials targeting HIF-1 have not been successful in the past. Keeping this finding in mind, targeting Vav1 may be the new approach to overcome hypoxic tumors.

## Supplementary Materials

The following are available online at https://www.mdpi.com/2072-6694/12/6/1374/s1, Figure S1: Whole blot images from Figure 1, Figure S2: Whole blot images from Figure 3, Figure S3: Whole blot images from Figure 4, Figure S4: Whole blot images from Figure 5.
