# Supplementary material for: Vav1 is Essential for HIF-1α Activation via a Lysosomal VEGFR1-Mediated Degradation Mechanism in Endothelial Cells
Source: Cancers (Basel). 2020 May 27;12(6):1374. doi: 10.3390/cancers12061374 (PMC7352305; doi:10.3390/cancers12061374)
Supplement: Supplementary file 1 [file cancers-12-01374-s001.pdf]

cancers

Vav1 is Essential for HIF-1α Activation via a Lysosomal VEGFR1-Mediated Degradation Mechanism in Endothelial Cells

Jaewoo Hong †,*, Yongfen Min †, Todd Wuest and P. Charles Lin *

Center for Cancer Research, National Cancer Institute, National Institutes of Health, Frederick, MD 21704, USA; yongfen.min@nih.gov (Y.M.); todd.wuest1@gmail.com (T.W.)
† These authors contributed equally.
* Correspondence: jaewoo.hong@nih.gov (J.H.); p.lin@nih.gov (P.C.L.)

Supplementary

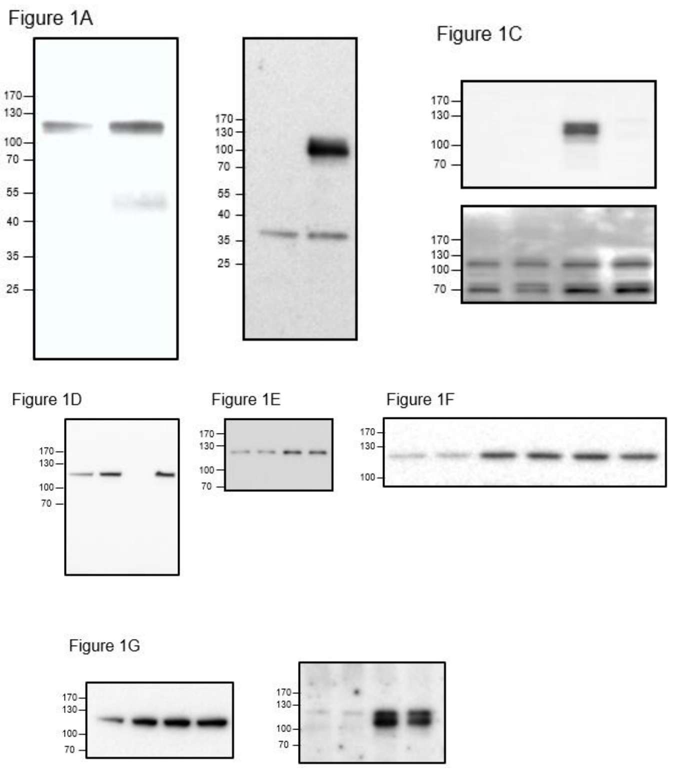

Figure S1. Whole blot images from Figure 1.

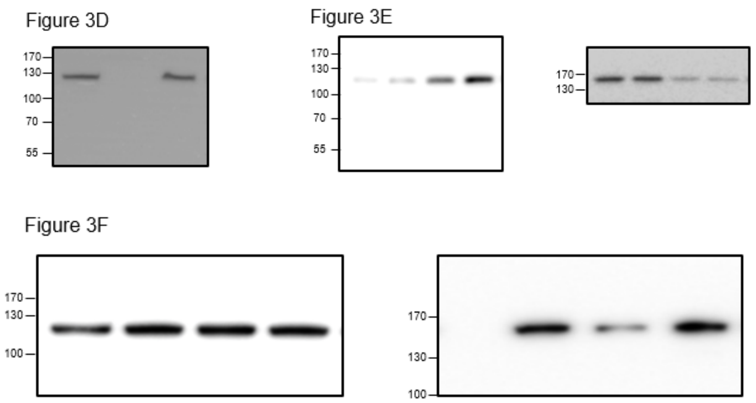

Figure S2. Whole blot images from Figure 3.

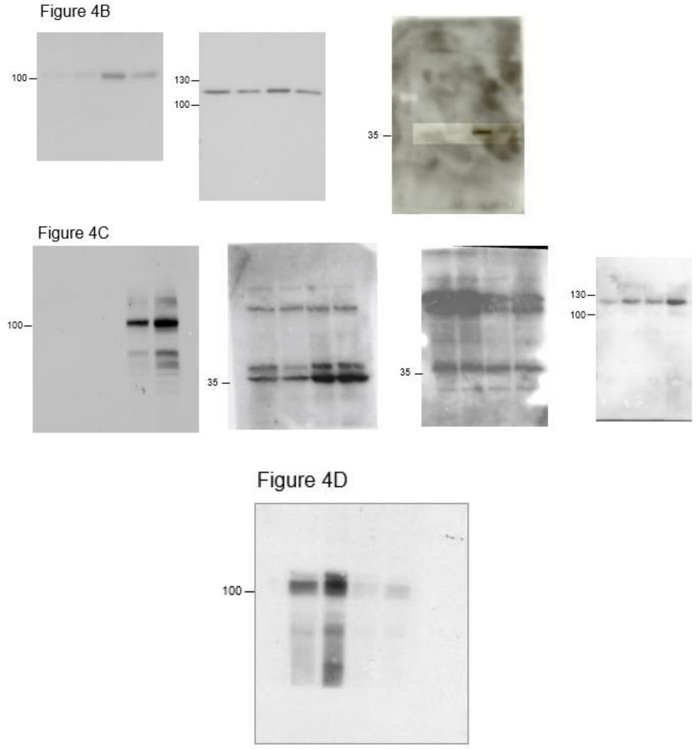

Figure S3. Whole blot images from Figure 4.

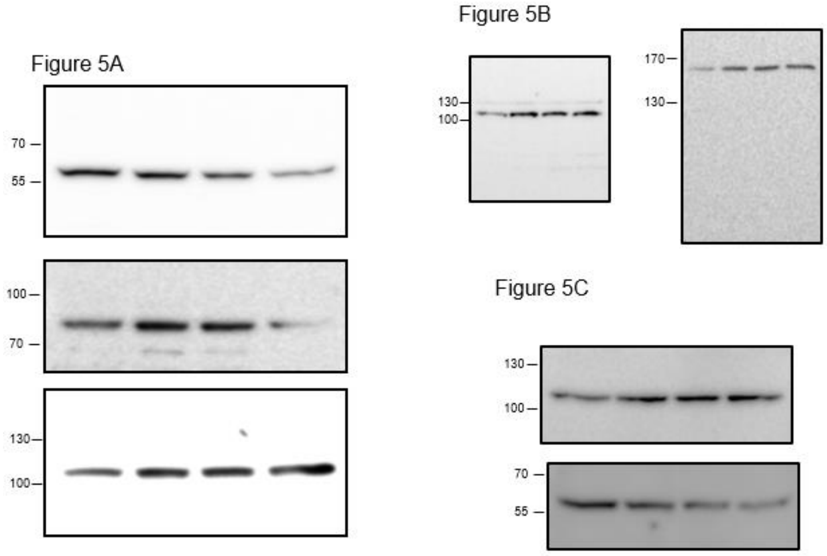

Figure S4. Whole blot images from Figure 5.
